# Impact Factors in Chinese Construction Enterprises’ Carbon Emission-Reduction Intentions

**DOI:** 10.3390/ijerph192416929

**Published:** 2022-12-16

**Authors:** Bo Wang, Chunyan Huang, Huaming Wang, Fangwei Liao

**Affiliations:** 1 School of Civil Engineering and Architecture, Southwest University of Science and Technology, Mianyang 621010, China; 2 School of Economics and Management, Southwest University of Science and Technology, Mianyang 621010, China; 3 School of Management, University of Science and Technology of China, Hefei 230026, China

**Keywords:** impact factors, construction enterprises, carbon emission-reduction intention, carbon peaking and carbon neutrality

## Abstract

The reduction in carbon emissions in the construction industry plays an important role in the realization of the goal of carbon peaking and carbon neutrality, especially in China. Construction enterprises’ personnel’s intentions to reduce their carbon emissions are closely related to low-carbon behavior. However, the research on the impact factors of carbon emission-reduction intention (CERI) is still limited. In order to understand the factors that affect the intentions of construction enterprises’ personnel to reduce carbon emissions, and to put forward valuable suggestions for reducing construction enterprises’ carbon emissions, it is necessary to explore the impact factors in carbon emission-reduction intention through empirical tests. This study adopted the theory of planned behavior (TPB) based on the three impact factors of behavioral attitude (BA), subjective norms (SNs) and perceived behavioral control (PBC), introduced the two potential impact factors of moral obligation (MO) and government supervision (GS), and also uses structural equation modeling (SEM) to test the impact factors in construction enterprises’ personnel’s carbon emission-reduction intention. The results show that BA, MO and GS have a significant positive impact on carbon emission-reduction intention (CERI). Among them, BA plays an intermediary role between MO and CERI, and BA and MO play a chain intermediary role between GS and CERI. The research conclusions will help to provide a theoretical reference for governments and construction enterprises to implement carbon emission-reduction development strategies and policies.

## 1. Introduction

According to the United Nations Framework Convention on Climate Change (UNFCCC)’s latest “Nationally Determined Contributions Synthesis Report”, countries around the world have strengthened their commitment to reduce greenhouse gas emissions, and put forward clear emission-reduction targets [1]. As the largest contributor to carbon dioxide emissions at present [2], China has strictly put forward the carbon reduction goal to “strive to achieve carbon peak by 2030 and carbon neutrality by 2060” [3]. As an important part of the global economy, the construction industry has the characteristics of high carbon emissions and high energy consumption [4]. The energy consumption of China’s buildings accounts for about 6.0% of global energy consumption, which is equivalent to the total energy consumption of the Middle East, or twice the consumption level of Africa, or the combined consumption of Japan and South Korea [5]. It is expected that by 2050, the energy consumption of the Chinese construction industry will continue to maintain a growth trend [6,7]; the carbon emission-reduction potential of its construction industry is much higher than that of most other high-carbon industries [8]. Therefore, paying close attention to the carbon emission-reduction intentions of construction enterprises’ personnel is of great significance to China’s ecological sustainable development and the achievement of carbon peak and carbon neutrality [9,10].

The carbon emission reduction of an enterprise is influenced by many factors. Among them, uncertainty over government behavior [11], the immaturity of low-carbon energy-saving technologies [12,13], the low efficiency of energy use [14,15,16] and difficulty of cost control [17,18] have become common problems in the process of the carbon emission reduction of construction enterprises. To alleviate the above problems, governments have launched active responses and incentive policies. Carbon emissions are macro-controlled by establishing green building markets [19], implementing carbon tax policies [20], promoting low-carbon sustainable building energy-saving technologies [21] and encouraging enterprises to develop renewable energy sources. These not only gradually become the main driving force for enterprises to carry out green practices [17,22], but also lay a good environmental and social foundation for the construction industry to implement carbon emission reduction faster and more effectively.

Theory and practice have shown that behavioral factors, such as perceived adaptive capacity [23] or motivation, are often more important than external resource constraints [24] in influencing pro-environment behavior. Therefore, scholars are gradually emphasizing these factors, which cause changes in enterprise personnel behavior, rather than exploring the impact of external conditions such as new technologies [25,26,27]. However, there are relatively few studies on the influence of construction enterprise personnel’s intentions to reduce carbon emissions. This paper introduces the theory of planned behavior (TPB) into the research on the carbon emission-reduction behavior of construction enterprises’ personnel, identifies the factors that may affect the intentions of construction enterprises’ personnel to reduce carbon emissions, establishes a structural equation model (SEM), and analyzes the key factors and influencing mechanisms that affect construction enterprises’ personnel’s carbon emission-reduction intentions. Next, the paper proposes management implications, which can provide references for the government and construction enterprises to improve their carbon reduction governance mechanisms to achieve carbon peak and carbon neutrality.

## 2. Literature Review

At present, most of the research on behavior and intentions in the scientific research community is based on the TPB [28,29]. The TPB suggests that personal intention is jointly affected by three factors: behavioral attitudes (BA), subjective norms (SNs) and perceived behavior control (PBC); personal intention directly affects behavior to a certain extent [30]. The TPB can not only predict the psychological drivers of a specific behavior, but also provide valuable insights for improving social and environmental sustainability [30,31,32]. Therefore, in recent years, the TPB has been widely used in various scientific fields, especially in environmental psychology, and it has been increasingly promoted as a key theory for predicting and promoting various pro-environmental behaviors [30,32,33]. For example, Lin (2013) [34] used the TPB to investigate the environmental-protection behaviors of Kaohsiung citizens in Taiwan in response to climate change, and found that citizens’ attitudes towards global warming affect their environmental-protection-behavior intentions. Yang et al. (2019) [28] analyzed the green-procurement behavior of developers and concluded that SNs and PBC have a significant impact on the intentions of developers to engage in green-procurement behavior. Therefore, this paper chooses the TPB as the theoretical basis for research on carbon-emission-reduction in the building field.

Increasing numbers of scholars choose to introduce potential influencing factors, such as moral obligation (MO), into the research field of environmental behavior to explain and predict specific behavior and intentions more comprehensively [25,29,35,36]. Dernbach and Brown (2009) [37] pointed out that no country can successfully deal with global climate change without the active participation of its citizens. If global climate change is viewed as a moral situation, and individuals or organizations have the responsibility to engage in energy-saving or emission-reduction behaviors, their intention to participate in energy saving and emission-reduction will also increase [35]. If an organization proves to have moral initiative, then it can take on the corresponding MO, and the reasons for the organization to fulfil its obligation to reduce greenhouse gas emissions increase [38,39]. Raats et al. (1995) [40] also believe that MO plays a strong role in predicting intention to engage in pro-environmental activities such as energy conservation and emission-reduction. Therefore, it is necessary to introduce MO as an independent predictor in the study of the carbon emission-reduction intentions of construction enterprises’ personnel.

As regulators and investors increasingly call for more climate-related corporate accountability, the importance of the environment is more prominent than ever, especially from the perspective of directors facing oversight responsibilities [41]. In recent years, the government of China has issued many environmental protection policies and regulations aimed at achieving the sustainable development of the country [19,20,21,42]. Because any violation may lead to sanctions, and compliance with the rules may be rewarded politically and economically, these policies and regulations greatly affect the behavioral decisions of managers and the business work of employees [42]. They are willing to carry out green activities in order to obtain government support and incentives [43]. The organizational efficiency and sustainable development of these energy-intensive enterprises have been further strengthened under the supervision of environmental policy [43,44]. All this shows that government supervision plays an important role in the environmental behavior and carbon emission-reduction intentions of construction enterprises’ personnel [45,46].

## 3. Hypothesis Development

### 3.1. Behavioral Attitude (BA)

Attitudes are the positive or negative feelings an individual holds about certain behavior, which have a significant impact on behavioral outcomes [47]. They can also be understood as an evaluation of behavioral outcomes based on rational choice [35]. Michalos et al. (2011) [48] believe that attitude is more influential than knowledge in determining sustainable development. If consumers are increasingly concerned about environmental sustainability, this naturally indicates that they will act in a more environmentally friendly way and have a greater intention to pay to support the environment [49]. When a person takes a more positive attitude towards mitigating global climate change, their likelihood of engaging in energy-saving or carbon-reducing behaviors increases [35]. In construction, contractors with a positive attitude to carbon reduction are usually more satisfied with waste-management behavior than those with a negative attitude [50]. We can infer that there is a significant positive association between BA and carbon emission-reduction intention (CERI) of construction enterprise personnel, which is also consistent with Ajzen’s TPB [51]. Therefore, hypothesis H1 was proposed:

**Hypothesis** **1** **(H1).** *BA has a significant positive impact on CERI*.

### 3.2. Subjective Norms (SNs)

Subjective norms (SNs) refer to the social pressure that participants feel about whether or not to engage in particular behaviors [47]. Social human behavior is not an independent process of individual choice, but is often influenced by social factors, such as the external environment or other groups [52]. This social pressure influences whether participants engage in certain behaviors [35]. According to the TPB, social pressure has a significant impact on the intentions of individuals to act, thereby affecting their actual behavior [47]. Some studies have pointed out that Chinese construction developers have begun to pay attention to the green development strategies adopted by their competitors and actively adjusted their own strategic planning [53]. It can be predicted that due to market pressure and competition, increasing numbers of enterprises will carry out green-procurement activities in the construction market in the future [28]. Therefore, hypothesis H2 was proposed:

**Hypothesis** **2** **(H2).** *SNs have a significant positive impact on CERI*.

### 3.3. Perceived Behavioral Control (PBC)

When deciding whether to engage in a specific behavior, the subject has the ability to perceive the difficulty of the behavioral goal, that is, the perception behavior [28]. Perceived behavior control (PBC) is the perceived degree of the subject’s control over the behavior in which it is engaged, which is jointly determined by perceived self-efficacy and perceived controllability [54], and reflects the subject’s past experience and expected obstacles. When the subject believes that the more resources and opportunities at his disposal, the fewer the expected obstacles, the stronger the PBC [47]. PBC can be further divided into internal and external dimensions. External-control perception leads to internal-control perception, and internal-control perception affects behavioral intention [55]. In construction enterprises, if developers do not have sufficient control over their environment and resources, they face significant resource constraints which affect their enthusiasm for green activities [28]. This shows that PBC has motivational implications for behavioral intentions. Therefore, hypothesis H3 was proposed:

**Hypothesis** **3** **(H3).** *PBC has a significant positive impact on CERI*.

### 3.4. Moral Obligation (MO)

The moral obligation (MO) in this paper refers to the perception of moral obligation towards environmental behavior. It refers to a person’s perception of whether it is morally right or wrong to engage in certain environmental behaviors [56]. Raats et al. (1995) [40] found that MO supports the theory of reasoned action. Generally, enterprises with a high sense of social responsibility consider MO when engaging in certain behaviors, such as carbon emission reduction [35]. This sense of MO drives enterprises’ personnel to actively participate in environmental practices, such as resource consumption and waste recycling, thereby reducing their impact on the environment [57,58]. Stern et al. (1998) [59] also believe that moral norms have an important impact on environmental behavior, and that ethically responsible enterprises’ personnel usually possess a positive low-carbon environmental philosophy or attitude, which in turn leads to the implementation of “responsible environmental behavior”. Therefore, it can be speculated that MO could significantly improve the predictive effects of CERI and BA. In light of this, the following hypotheses, H4, H5 and H6 were proposed:

**Hypothesis** **4** **(H4).** *MO has a significant positive impact on CERI*.

**Hypothesis** **5** **(H5).** *MO has a significant positive impact on BA*.

**Hypothesis** **6** **(H6).** *BA plays an intermediary role between MO and CERI*.

### 3.5. Government Supervision (GS)

With regulators and investors increasingly demanding more climate-related corporate accountability, the importance of the environment is more prominent than ever [41]. Generally, the government or authorities strengthen environmental supervision and put forward more environmental requirements so that the enterprise personnel will feel the pressure gradually increasing, and their attitude to carbon reduction will improve and strengthen [14,38]. At the same time, GS is often accompanied by government oversight support, and this oversight support behavior has a strong impact on employees’ willingness to promote eco-initiatives that will improve environmental performance and reduce natural environmental impacts [60]. This is one of the reasons why companies affected by environmental pressures are more proactive in dealing with environmental issues [61]. In addition, when its level is appropriate, environmental supervision can also play an important role in increasing corporate environmental responsibility [62]; corporate social responsibility is closely related to the ethical behavior of organizations [63]. It can be inferred that GS, as an important predictor of corporate social responsibility [64], can have a direct impact on strengthening corporate MO. Therefore, the following hypotheses were proposed:

**Hypothesis** **7** **(H7).** *GS has a significant positive impact on CERI*.

**Hypothesis** **8** **(H8).** *GS has a significant positive impact on SNs*.

**Hypothesis** **9** **(H9).** *GS has a significant positive impact on MO*.

**Hypothesis** **10** **(H10).** *MO plays an intermediary role between GS and CERI*.

**Hypothesis** **11** **(H11).** *BA and MO play a chain mediating role between GS and CERI*.

**Figure 1:**
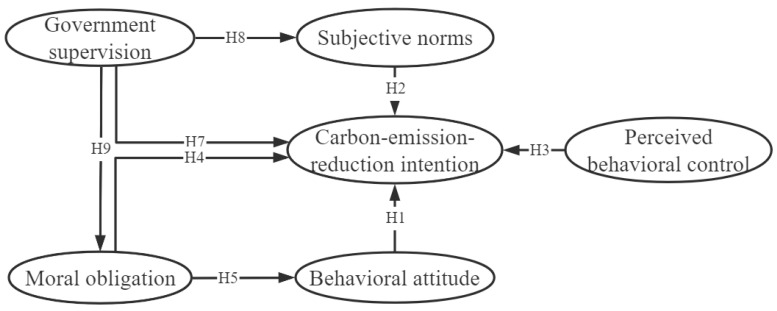
Theoretical model.

The initial theoretical model is shown in Figure 1.

## 4. Research Design

### 4.1. Questionnaire Design and Variable Measurement

In order to ensure the reliability and validity of the questionnaire used in this study, it developed the measurement of each factor on the basis of the major literature and adjusted it according to the research background. The questionnaire consisted of two parts. The first part was the basic information of the respondents, including the type of enterprise, the period of establishment of the enterprise, the nature of the enterprise, the size of the enterprise, the management level and the working years of the interviewee. The second part was the measurement of six variables, with a total of 23 questions. All items were measured using the Likert-scale method (1 = strongly disagree, 5 = strongly agree).

CERI mainly referred to the scale of Wang et al. (2019) [25] and Wu et al. (2017) [29], and a total of three items were set: “Would you like to participate in low-carbon emission-reduction training and education?”; “Would you prefer to use clean energy and green building materials?”; and “Do you support the government to open carbon market transactions?”. The higher the score of the item, the stronger the intention of enterprises’ personnel to carry out carbon emission-reduction activities. In this paper, the structure showed good reliability (Cronbach’s α = 0.724).

BA mainly referred to the scales used by Wu et al. (2017) [29] and Ajzen (2006) [65], and a total of four items were set. This paper mainly explores the attitudes of construction enterprises’ personnel towards carbon-emission-reduction from the perspectives of “social sustainability”, “enterprise brand effect”, “double-carbon goals”, and “environmental quality improvement”. The higher the score of each item, the more positive the enterprise personnel’s attitude towards carbon-reduction actions. In this paper, the structure shows good reliability (Cronbach’s α = 0.844).

SNs referred to the social pressure that construction enterprises employees feel when undertaking carbon emission-reduction activities. This variable also mainly adopts the scale used by Wu et al. (2017) [29] and Ajzen (2006) [65], and a total of 4 items were set. It mainly involved the social expectations or pressures of “public opinion”, “enterprise projects”, “potential customers”, and “family and friends”. In this paper, the structure showed good reliability (Cronbach’s α = 0.822).

PBC reflected the resource status of construction enterprises employees when they carry out carbon emission reductions. The design of the perceptual behavior control scale mainly referred to the scales used by Wu et al. (2017) [29] and Ajzen (2006) [65]. PBC mainly expressed whether the construction enterprise had enough “funds”, “technology”, “time”, “experience”, etc., and a total of 5 items were set in the scale. In this paper, the structure showed good reliability (Cronbach’s α = 0.847).

MO referred to the used by Chen (2016) [35], and combined the characteristics of “carbon peak“, “carbon neutralization”, and carbon emission reduction. A total of four items were set, such as “whether there is an obligation to reduce carbon emissions and protect the environment”, “whether there is an obligation to adopt clean energy”, etc. The higher the score of the item, the stronger the person’s perception of the MO to reduce carbon emissions. In this paper, the structure showed good reliability (Cronbach’s α = 0.766).

GS referred to the scales used by Wang et al. (2019) [25], Wu et al. (2017) [29] and Du et al. (2021) [36]. A total of three items were set, which involved the question of whether the government has “clear emission reduction policies”, “special regulatory departments”, and “perfect reward and punishment measures”. The higher the score of each item, the stronger the regulatory role of government departments in the carbon emission-reduction behavior of construction enterprises. In this paper, the structure showed good reliability (Cronbach’s α = 0.762).

### 4.2. Data Collection

In this paper, data were obtained by issuing questionnaires to construction enterprises’ personnel in China. The construction industry is the lifeblood of the national economy; it has made important contributions in promoting economic growth, relieving employment pressure and improving people’s quality of life in China. At the same time, the construction industry is also facing very serious problems, such as high energy consumption, high carbon emissions and high environmental pollution. Therefore, accelerating the carbon emission reduction of construction enterprises is of great significance to improving China’s environmental governance and achieving China’s “dual carbon” goal.

To ensure the reliability and validity of the scale, the data collection of this research was divided into two stages. The first stage was pre-testing, which began in July 2021 and ended in August 2021; it used professors, associate professors, doctors, and corporate alumni in the relevant fields of the authors’ university as the survey respondents. A total of 75 questionnaires were distributed, and 71 valid questionnaires were obtained. All items had good discrimination through independent sample T-test, the Cronbach α value of all variables were greater than 0.700, and the factor loadings of the rotating component matrix were all greater than 0.600. This was in line with the expectations of the questionnaire design; therefore, the questionnaire could be formally distributed. The second stage was the formal test, which began in September 2021 and ended in November 2021. On one hand, questionnaires were filled out on site, and if the respondents were unclear about any of the questions, they could ask and receive answers on the spot, which ensured the validity of the questionnaire. On the other hand, online questionnaires were published on an internet platform and professionals in the construction field were invited to fill them in by publishing links to the questionnaires on professional fora related to construction. The online questionnaires had no geographical location or time constraints, and were efficient and cost effective [66]. A total of 483 questionnaires were collected, and the questionnaires with unqualified content were screened. Consequently, there were 333 available questionnaires and the rate of valid questionnaires was 68.9%.

### 4.3. Research Steps and Methods

The use of SEM in environmental sciences has increased rapidly in the last decade [67]. It is mainly used to examine the relationships between continuous or discrete independent variables and dependent variables [68]. This involves the theoretical framework of the hypothesis, the collection of variable data, and the verification of the rationality and correctness of the set structural relationship or model hypothesis. Therefore, SEM, as a comprehensive multivariate statistical technique, was chosen by the authors as the primary data model for this study.

The specific steps and methods of this paper are as follows. The first was a characteristic analysis of sample data. The basic characteristics of the collected questionnaire data were analyzed. The second was common method bias. Referring to the study by Podsakoff et al. (2003) [69], Harman’s single-factor method was used to test whether there was a common method bias in the scale. The third was a test on reliability and validity. In order to ensure the reliability and accuracy of the scale in this study, SPSS (Version 23.0, IBM Corp., Armonk, NY, USA) and AMOS (Version 26.0, IBM Corp., Armonk, NY, USA) were used for the reliability and validity test and model-fitting analysis of the scale. The fourth was hypothesis testing and mediating-effect testing. The bootstrap method was used to test whether MO and BA had mediating effects, and the factors influencing the effects were analyzed. The fifth was a discussion and summary. The research results of this paper are summarized, the relevant guidelines are put forward and the deficiencies of the research and the prospects of future research are explained.

## 5. Analysis and Results

### 5.1. Characteristic Analysis of Sample Data

The analysis of the data characteristics of this scale is shown in Table 1. The respondents came from different types of construction enterprises, including housing construction (36%), civil engineering (28.5%), construction and installation (12.6%) and building decoration and decoration (8.7%). Their positions in the enterprises are senior manager (7.8%), middle manager (15.9%), grassroots manager (31.8%), professional and technical personnel (32.7%) and others (11.7%). Most of the respondents worked in construction enterprises that had a relatively good understanding of carbon emission reduction in the construction industry. Their answers can be said to truly reflect the intentions of construction enterprises’ personnel to implement carbon emission reduction.

### 5.2. Common Method Biases Test

When data are collected from a single source at the same time point, the problem of common method bias (CMB) may occur, which reduces the effectiveness of the study. In this study, the Harman’s single-factor test proposed by Podsakoff et al. (2003) [69] was used to test for common method bias using SPSS 23.0 software. The results showed that the variance explaining the rate of the first factor was 36.39%, which was lower than the threshold of 40% [69], indicating that there was no common method bias in this study.

### 5.3. Reliability and Validity Test

Through the analysis of SPSS 23.0, the KMO value was 0.926 > 0.8, and the Chi-square value of Bartlett’s spherical test was 3505.162, *p* < 0.01, indicating that the data passed the significance test and were suitable for factor analysis and validity test. The Cronbach’s alpha value of the sample was 0.924, indicating that the data was suitable and had good reliability. The validity analysis is from two aspects: convergence validity and discriminant validity. Among them, the main reference index of convergent validity is AVE, which represents the comprehensive explanatory ability of potential variables to all measured variables. In Table 2, although the AVE values of some factors are not greater than 0.5, according to the study of Purnomo (2017) [70], it is recommended that the aggregation validity of factors is acceptable with AVE values of less than 0.5 but C.R. greater than 0.7, which shows that the measurement indices of each factor of the questionnaire were well extracted and that the convergence effect is acceptable. The main indicator of discriminant validity is the square root of AVE. If the arithmetic square root of AVE is greater than the absolute value of the correlation coefficient between the potential variables, indicating that there is a difference between the potential variables, then the discriminant validity is high. In Table 3, it can be seen that the square root value of the AVE of each factor in the scale is greater than the correlation square of other variables, indicating that the model has good discriminant validity.

### 5.4. Model Modification and Verification

It can be seen from Table 4 that the data fitting of the initial model is poor. Combined with the initial model analysis results, as shown in Table 5, it can be seen that there are some paths with insignificant *p* values. Therefore, it is necessary to delete unimportant paths and modify the initial model. The path with the highest *p* value and the least significant is deleted first. PBC has no significant path to carbon emission-reduction intention (*p*_3_ = 0.956 > 0.05), that is, PBC cannot have a direct positive impact on behavioral intention. Therefore, the constructs of the PBC are deleted to create a new model. After a similar revision process, the paths of SNs, etc. were removed from CERI. The final modified structural model is obtained. 

**Figure 2:**
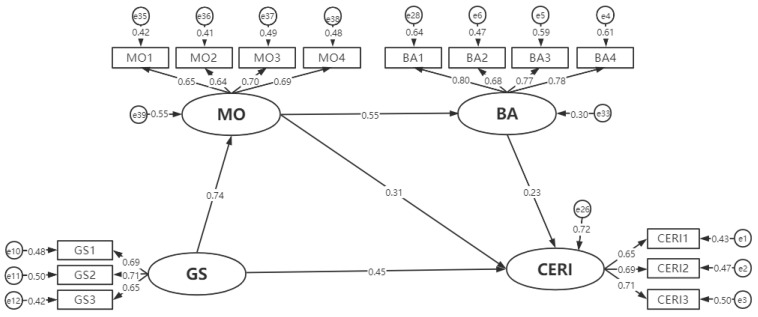
Normalized factor loadings and path factors for SEM. Notably, the number between latent variables indicates standardized path factors. The number from latent variables to measured variables indicates normalized factor loadings.

The modified model meets the goodness of fit standard and is superior to the initial model, as shown in Table 6, without further modification. The analysis results of the modified model are shown in Table 7: BA (***p***_1_ < 0.01), MO (***p***_4_ < 0.01) and GS (***p***_7_ < 0.001) have significant positive effects on CERI, supporting the hypotheses H1, H4 and H7. In addition, the test also found that MO significantly increased BA (***p***_5_ < 0.001), supporting Hypothesis H5. GS also had a significant positive effect on SNs and MO (***p***_8_ < 0.001, ***p***_9_ < 0.001), indicating that Hypothesis H8 and H9 were confirmed. As can be seen from Table 5, PBC (***p***_3_ > 0.05) and SNs (***p***_2_ > 0.05) had no significant effects on CERI. Therefore, we can suppose that H2, H3 are rejected. Figure 2 is the normalized SEM path diagram after correction.

### 5.5. Mediating Effect

In this study, AMOS 26.0 software and the bootstrap method were used to test the mediation effect by repeated sampling 2000 times. The results are shown in Table 7 and Table 8. The upper and lower confidence intervals of the two mediating paths H6: MO → BA → CERI and H11: GS → MO → BA → CERI do not include 0, indicating that the mediating effect is significant. However, the mediating path of the path H10: GS → MO → CERI is not significant; thus, there is no mediating effect.

Table 9 shows the effects of each factor. According to the contribution degree of each factor in the total effect or direct effect, GS, MO and BA are in order from high to low. The total impact on carbon emission-reduction intention is 0.773, 0.435 and 0.228, respectively, and the direct impact is 0.451, 0.309 and 0.228, respectively. The indirect effect of GS is 0.322 through the chain mediation between MO and BA, MO has an indirect effect of 0.125 mediated by BA.

## 6. Conclusions and Discussion

### 6.1. Conclusions

Based on the theory of planned behavior (TPB), this study introduced two potential impact factors, moral obligation (MO) and government supervision (GS), and used the structural equation model (SEM) to test the impact factors of construction enterprises’ personnel’s carbon emission-reduction intention.

MO and GS are important factors affecting the behavioral attitudes and intentions of employees in construction enterprises’ personnel to reduce carbon emissions. Therefore, the impact of MO and GS should not be ignored in the planning and design of future corporate carbon emission-reduction strategies [35]. These can not only improve the behavioral intentions of construction enterprises’ personnel in terms of carbon emission-reduction, but also increase the social responsibility and improve the environmental ethics of enterprises, thereby enhancing their competitive advantage.

Through the mediation effect analysis, the chain mediating role of MO and BA in GS and CERI and the mediating role of BA in MO and CERI were verified. This indicates that with the strengthening of the government or other regulatory authorities, construction enterprise staff tend to perceive that the stronger MO, the more positive the attitude to carrying out carbon emission-reduction actions, and thus a higher CERI is formed. Therefore, regulatory authorities should properly strengthen environmental supervision and strengthen the concept of the low-carbon environmental moral obligation of construction personnel, as this has a positive impact on the environmental management strategy of construction enterprise personnel.

At the same time, this study found that PBC and SNS did not have a significant impact on the carbon emission-reduction intentions of construction enterprises’ personnel. One reason for this is that excessive PBC is not conducive to the prediction of behavioral intention [71]. Trafimow and Duran (1998) [72] believe that when PBC is too high, its predictive power for behavioral intention becomes irrelevant [73]. Meanwhile, higher PBC tends to reduce the relative importance of SNs in intention prediction [74]. On the other hand, the strength of PBC intention also depends on differences in familiarity with behaviors [71,75,76]. Carbon peak and carbon neutralization are the newly proposed low-carbon strategic goals. Consequently, the ability of PBC to predict the intention of carbon emission reduction is not necessarily significant.

### 6.2. Theoretical Contribution

The theoretical contribution of this paper lies in the following points: 

Firstly, this study introduced the TPB into the study of the carbon emission reduction field of construction enterprises and confirmed that BA has a positive role in encouraging carbon emission reduction among construction enterprises’ personnel. This not only broadens the application scope of the TPB, but also compensates for the lack of attention paid to the carbon emission-reduction intentions of construction enterprises’ personnel in previous studies.

Secondly, this paper added MO and GS as two potential impact factors, and found that MO and GS not only have a direct impact on the carbon emission-reduction intentions of construction enterprises’ personnel, but also have a significant indirect impact on carbon emission-reduction intentions by influencing BA. This theoretically enriches the research on the influencing factors of TPB and the carbon emission-reduction intentions of construction enterprises’ personnel. This paper also confirmed the important role of MO and GS in improving intentions to reduce carbon emissions among construction enterprises’ personnel, which indicated that construction enterprises should carefully consider the impact of both in future environmentalnprotection decisions.

In general, this study provides a new dimension for the study of enterprises’ carbon emission-reduction intentions, and lays a new theoretical foundation for the study of construction enterprises employees’ carbon emission-reduction behavior and sustainable development.

### 6.3. Management Insights

This study can provide references for the government and construction enterprises to improve their governance mechanisms to achieve carbon peak and carbon neutrality.

Firstly, the government can strengthen the supervision and governance functions of carbon emission reduction in construction enterprises. The government can continue to strictly follow the principle of “paying for pollution” and formulate punishment levels for enterprises with carbon discharge that is indiscriminate or in large quantities and enterprises that do not implement carbon emission-reduction policies, such as increasing fines or ordering suspension of business, so as to promote carbon emission reduction to those working in the construction sector. At the same time, the government will improve the emission-reduction subsidy measures and policies, and focus on supporting enterprises that have difficulties in implementing carbon emission reduction, so as to avoid a situation in which enterprises will be reluctant to adopt carbon emission reduction due to continuous increases in cost and declines in efficiency which will lead to the failure of GS.

Secondly, construction enterprises can combine carbon-reduction culture with the cultivation of environmental MO among enterprise personnel. In recent years, the construction of a low-carbon culture among enterprises has been developed, and is often discussed, but the consideration of the cultivation of MO among enterprise employees for environmentally sustainable development has not been undertaken in depth. Therefore, in order to improve the overall carbon-reduction initiative of construction enterprises’ employees, it is necessary for construction enterprises to strengthen their carbon-reduction culture, focusing on the cultivation of an awareness of environmental MO among corporate personnel in order to further strengthen the momentum behind the overall low-carbon and carbon-reduction intentions of construction enterprises’ employees.

Thirdly, this study strengthens the awareness of carbon emission reductions among construction personnel and the promotion of the concept in general. During the survey, this study found that most of the respondents stated that they lacked a basic understanding of “carbon peak” and “carbon neutrality”, and their awareness of carbon reduction was relatively insufficient. The primary focus of construction enterprises’ employees is always on economic interests, rather than on the ecological environment. Moreover, most construction workers do not realize that the essence of achieving “carbon peak” and “carbon neutrality” is energy saving and carbon reduction. Therefore, it is necessary to continue to promote the low-carbon development of construction enterprises and awareness of this concept among their personnel in the future.

### 6.4. Deficiencies and Prospects

As an exploratory empirical study, this study has certain limitations. Firstly, as it focuses on the impact of GS and MO on carbon emission-reduction among construction enterprises’ personnel, the research process may have ignored the influence of other factors, such as carbon emission-reduction technology and investors. Future research should consider the possible influencing factors more comprehensively in order to further enrich the research’s content and broaden its vision. Secondly, this study focuses on the carbon reduction intentions of construction enterprises’ personnel, which is different from the carbon reduction intentions of construction enterprises. Future research can be conducted by introducing theories related to collective organizational behavior to strengthen the theoretical basis of the study, or by considering construction enterprises as the main subject of the study. Thirdly, enterprises’ personnel with different ownership models or scales have different abilities to receive and respond to the new policy goals. Researchers can compare and analyze the influencing factors behind the carbon emission-reduction intentions of different types of enterprise and further explore the internal mechanisms that affect the carbon emission-reduction behavior of construction enterprises’ personnel.

## Figures and Tables

**Table 1:** Statistical analysis of respondent sample.

Variables	Options	Frequency	Percentage (%)	Cumulative Percent (%)
Type of enterprise	Housing construction	120	36	36
	Civil engineering	95	28.5	64.6
	Building installation	42	12.6	77.2
	Building decoration	29	8.7	85.9
	Other	47	14.1	100
Time of enterprise establishment	3 years or below	34	10.2	10.2
	4–5 years	49	14.7	24.9
	6–10 years	79	23.7	48.6
	11–20 years	73	21.9	70.6
	21 years or more	98	29.4	100
Nature of enterprise	State-owned enterprise	128	38.4	38.4
	Sole state-owned enterprise	16	4.8	43.2
	Joint-stock enterprise	61	18.3	61.6
	Private enterprise	115	34.5	96.1
	Joint venture	7	2.1	98.2
	Other	6	1.8	100
Size of enterprise	300 persons or below	113	33.9	33.9
	300–499 persons	42	12.6	46.5
	500–999 persons	54	16.2	62.8
	1000–1999 persons	45	13.5	76.3
	2000–4999 persons	23	6.9	83.2
	5000 persons or more	56	16.8	100
Respondent distribution	Senior managers	26	7.8	7.8
	Middle managers	53	15.9	23.7
	Grassroots managers	106	31.8	55.6
	Professionally skilled worker	109	32.7	88.3
	Other	39	11.7	100
Respondent working years	5 years or below	171	51.4	51.4
	6–10 years	78	23.4	74.8
	11–15 years	55	16.5	91.3
	16–20 years	16	4.8	96.1
	21–25 years	8	2.4	98.5
	26 years or more	5	1.5	100

**Table 2:** Reliability and validity test.

Latent Variable	Item	Normalized Factor Loadings	Z-Values	S.E.	AVE	C.R.	Cronbach’s Alpha
Carbon emission-reduction intention (CERI)	CERI1	0.651	-	-	0.466	0.723	0.724
	CERI2	0.682	9.951	0.101			
	CERI3	0.712	10.253	0.106			
Behavioral attitude (BA)	BA1	0.769	-	-	0.578	0.845	0.844
	BA2	0.764	13.794	0.071			
	BA3	0.691	12.386	0.07			
	BA4	0.81	14.635	0.07			
Subjective norms (SNs)	SN1	0.729	-	-	0.526	0.816	0.822
	SN2	0.691	11.959	0.083			
	SN3	0.733	12.678	0.08			
	SN4	0.736	12.745	0.081			
Perceived behavioral control (PBC)	PBC1	0.711	-	-	0.525	0.847	0.847
	PBC2	0.727	12.163	0.081			
	PBC3	0.736	12.302	0.08			
	PBC4	0.701	11.759	0.085			
	PBC5	0.747	12.473	0.085			
Moral obligation (MO)	MO1	0.668	-	-	0.461	0.72	0.766
	MO2	0.706	10.58	0.105			
	MO3	0.662	10.062	0.11			
	MO4	0.653	9.954	0.102			
Government supervision (GS)	GS1	0.705	-	-	0.478	0.733	0.762
	GS2	0.691	11.193	0.09			
	GS3	0.65	10.589	0.089			

**Table 3:** Pearson correlation and AVE root value (*N* = 333).

	CERI	BA	SNs	PBC	MO	GS
**CERI**	0.683					
**BA**	0.466	0.760				
**SNs**	0.528	0.583	0.725			
**PBC**	0.425	0.357	0.603	0.725		
**MO**	0.554	0.428	0.456	0.423	0.679	
**GS**	0.545	0.338	0.508	0.530	0.520	0.691

Note: The diagonal number is the root value of the factor AVE.

**Table 4:** Initial model-fitting index.

Index	CMIN/DF	GFI	AGFI	RMSEA	SRMR	NFI	NNFI	CFI
Condition	<3	>0.95	>0.90	<0.07	<0.08	>0.95	>0.95	>0.95
Result	2.687	0.868	0.838	0.071	0.1829	0.818	0.877	0.876

**Table 5:** Initial hypothesis test results.

Hypothesis	Path	Estimate	S.E.	T Value	*p* Value	Results
H1	BA → CERI	0.287	0.143	2.006	*	Accept
H2	SNs → CERI	0.101	0.09	1.122	0.262	Reject
H3	PBC → CERI	−0.003	0.059	−0.055	0.956	Reject
H4	MO → CERI	0.321	0.133	2.411	*	Accept
H5	MO → BA	0.737	0.093	7.914	***	Accept
H7	GS → CERI	0.411	0.149	2.758	**	Accept
H8	GS → SNs	0.816	0.092	8.884	***	Accept
H9	GS → MO	0.749	0.084	8.935	***	Accept

Note: ***, **, and * indicate *p* < 0.001, *p* < 0.01, *p* < 0.05, respectively.

**Table 6:** Modified model-fitting index.

Index	CMIN/DF	GFI	AGFI	RMSEA	SRMR	NFI	NNFI	CFI
Condition	<3	>0.95	>0.90	<0.07	<0.08	>0.95	>0.95	>0.95
Result	1.555	0.951	0.929	0.041	0.036	0.936	0.976	0.976

**Table 7:** Modified hypothesis test results.

Hypothesis	Path	Estimate	S.E.	T Value	*p* Value	Results
H1	BA → CERI	0.186	0.058	3.227	**	Accept
H4	MO → CERI	0.318	0.119	2.661	**	Accept
H5	MO → BA	0.691	0.09	7.655	***	Accept
H7	GS → CERI	0.443	0.11	4.022	***	Accept
H9	GS → MO	0.71	0.084	8.489	***	Accept
H6	MO → BA → CERI	0.125	0.066	2.146	**	Accept
H10	GS → MO → CERI	0.229	0.134	1.729	0.075	Reject
H11	GS → MO → BA → CERI	0.093	0.048	2.324	**	Accept

Note: ***, **, and * indicate *p* < 0.001, *p* < 0.01, *p* < 0.05, respectively.

**Table 8:** Mediating effect.

	Bias-Corrected Percentile Method				Percentile Method				
	Estimate	Lower	Upper	*p* Value	Estimate	Lower	Upper	*p* Value	Results
Std. H6	0.125	0.037	0.286	0.006	0.125	0.035	0.286	0.006	Accept
Std. H10	0.229	−0.019	0.493	0.075	0.229	−0.041	0.471	0.102	Reject
Std. H11	0.093	0.026	0.209	0.006	0.093	0.026	0.208	0.006	Accept

**Table 9:** The effect of various factors on CERI.

Influence Path	Direct	Indirect	Total
BA → CERI	0.228	-	0.228
MO → CERI	0.309	0.125	0.435
GS → CERI	0.451	0.322	0.773
MO → BA	0.550	-	0.550
GS → BA	-	0.408	0.408
GS → MO	0.742	-	0.742

## Data Availability

Not applicable.
